# The effect of propofol on chemosensitivity of paclitaxel in cervical cancer cells

**DOI:** 10.1002/cam4.6064

**Published:** 2023-05-10

**Authors:** Yanshan Jin, Shangdan Xie, Bo Sheng, Mei Chen, Xueqiong Zhu

**Affiliations:** ^1^ Center for Uterine Cancer Diagnosis & Therapy Research of Zhejiang Province, Department of Obstetrics and Gynecology The Second Affiliated Hospital of Wenzhou Medical University Zhejiang China; ^2^ Department of Obstetrics and Gynecology Taizhou Women and Children's Hospital of Wenzhou Medical University Zhejiang China

**Keywords:** cervical cancer, paclitaxel, propofol

## Abstract

**Background:**

Propofol is a drug with potential anticancer effect. This study aimed to explore the effect of propofol on chemosensitivity of cervical cancer cells to paclitaxel.

**Methods:**

HeLa and CaSki cells were selected for drug experiments. Cell viability was evaluated via CCK‐8 assay, and the combination index (CI) was calculated by CompuSyn software. A clinically relevant concentration and IC30 of propofol were selected in combination with 5 nM paclitaxel. BrdU incorporation, transwell, and flow cytometry assays were utilized to evaluate cell proliferation, migration, invasion, and apoptosis. The expression of β‐tubulin, stathmin 1, and GAPDH proteins was evaluated by Western blot. The stathmin 1 cDNA plasmid was used to establish stathmin 1‐overexpressing CaSki cells.

**Results:**

At clinically relevant concentrations (0–80 μM), propofol did not affect cancer cell viability, but high concentrations (100–800 μM) reduced cell viability. The CI values of propofol with IC30 (200 μM in HeLa; 400 μM in CaSki) combined with 5 nM paclitaxel were <1. The effect of propofol with IC30 combined with paclitaxel on cell proliferation, migration, invasion, and apoptosis were stronger than individual effect, while 30 μM propofol had no effect. The Western blot results showed 30 μM propofol did not affect β‐tubulin and stathmin 1 expression in cells, although paclitaxel upregulated β‐tubulin expression while downregulating stathmin 1 expression. Compared with paclitaxel alone, cotreatment with propofol at its IC30 and paclitaxel decreased stathmin 1 expression but had no effect on β‐tubulin expression. High stathmin 1 expression weakened the effect of paclitaxel on cell viability and apoptosis, while propofol partially reversed these effect.

**Conclusion:**

Propofol at clinically relevant concentrations had no effect on the malignant biological behaviors of cervical cancer cells, while propofol at high concentrations decreased.Propofol with IC30 and paclitaxel had synergetic effect on cancer cells through a reduction in stathmin 1 expression.

## 1 INTRODUCTION

Propofol (2,6‐diisopropylphenol), a popular intravenous anesthetic, is distinguished by its stable induction of anesthesia and rapid reversal and is widely applied in resection surgery for malignant tumors^1^ and in various outpatient applications to allow painless operations. Propofol‐induced anesthesia usually occurs at a blood concentration of 4–6 mg/L, which is equivalent to 22–34 μM.^2^ Accumulating research has demonstrated that propofol can suppress the malignant biological behaviors of various cancer cells via many signaling pathways.^3^, ^4^ In addition, numerous studies have demonstrated that propofol reduces the resistance of cancer cells to many chemotherapeutic drugs.^5^, ^6^ For instance, propofol could increase the cytotoxicity of cisplatin in liver cancer.^5^ It has been suggested that propofol might enhance the effect of chemotherapeutic drugs on tumors.

Cervical cancer is a kind of cancer that is a serious threat to the lives of women. Although HPV vaccination reduces the incidence of cervical cancer, there were still an estimated 4290 deaths from cervical cancer in the United States in 2021.^7^ Surgery is the standard therapy for cervical cancer patients with early‐stage disease, while chemotherapy is used in patients with postoperative recurrence and in postoperative treatment of patients with advanced disease.^8^, ^9^, ^10^ Paclitaxel, a common anticancer drug, is a first‐line chemotherapy for advanced, metastatic, and recurrent cervical cancer.^11^ However, the emerging phenomenon of paclitaxel resistance might contribute to the poor prognosis of patients.^12^ A study showed that propofol enhanced the lethal effect of paclitaxel on prostate cancer cells.^6^ Studies concentrating on the influence of propofol on the cytotoxicity of paclitaxel in cervical cancer remain rare. Therefore, our research aimed to explore the effect of propofol at clinically relevant concentrations on the malignant behaviors of cervical cancer cells. Then, we analyzed the effect and potential mechanism of the combination of propofol at its IC30 and paclitaxel on the viability, proliferation, metastasis, and apoptosis of cervical cancer cells to assess the influence of propofol on the cytotoxicity of paclitaxel.

## 2 MATERIALS AND METHODS

### 2.1 Cell culture

HeLa and CaSki cell lines were provided by Shanghai Cell Biology Medical Research Institute. HeLa cells were cultured in DMEM (Gibco, USA), and CaSki cells were cultured in RPMI‐1640 medium (Gibco, USA). Both media were supplemented with 10% FBS (Gibco, USA) and 1% penicillin–streptomycin antibiotic solution. HeLa and CaSki cell lines were incubated in a humidified environment of 5% CO_2_/95% air at 37°C.

### 2.2 Reagents and antibodies

Propofol (Sigma, USA) was stored at room temperature, and two series of various concentrations of propofol (clinically relevant concentrations: 0, 5, 10, 20, 40, and 80 μM; high concentrations: 0, 100, 200, 400, 600, and 800 μM) were applied in the cell viability assay. Paclitaxel (MedChemExpress, USA) was stored at −80°C. The diaminobenzidine (DAB) substrate kit was obtained from Zhongshan Goldenbridge (China). The primary antibodies utilized were as follows: rabbit anti‐β‐tubulin (1:2000, ABclonal, AC008, China), rabbit anti‐stathmin 1 (1:1000, Abcam, ab52630, USA) and rabbit anti‐GAPDH (1:2000, CST, #5174, USA). The secondary antibody was a horseradish peroxidase‐conjugated goat anti‐rabbit antibody (1:2000, Biosharp, USA).

### 2.3 Cell counting kit‐8 (CCK‐8) assay

Cells (4000/well) were dispensed into 96‐well plates. After cell attachment, the cells were treated with the above two series of propofol concentrations for 24 or 48 h. For paclitaxel treatment, cells were cultured with a series of concentrations of paclitaxel (0, 2.5, 5, 10 nM) for 48 h. Afterward, 10 μL of CCK‐8 solution was added to each well and incubated for 2 h, and cell viability was assessed by measuring the OD_450_ values with a microplate reader (Thermo, USA). Cell viability was quantified via the listed equation:

$\%\text{Cell Viability}=\frac{\text{Absorbance individual test group}-\text{Absorbance blank group}}{\text{Absorbance control group}-\text{Absorbance blank group}}$. Then, the IC30 of propofol in HeLa and CaSki cells was selected for use in combination with the above concentrations of paclitaxel to evaluate the interaction of the two drugs. The experiment was repeated three times.

### 2.4 Drug combination effect assay

The potential synergistic or antagonistic effect of propofol and paclitaxel was analyzed by the Chou–Talalay method.^13^ The values of the combination index (CI) were computed via CompuSyn software. The relationship between the CI value and drug–drug interaction effect is as follows: CI <0.90, synergism; CI = 0.9 ~ 1.1, additive; CI >1.1, antagonism. The CI values were conducive to determine the final concentration of paclitaxel in further experiments. The subsequent proliferation and apoptosis assays were divided into two parts. In one part, the effect of 30 μM propofol on paclitaxel efficacy was explored, and in the other part, the effect of propofol at its IC30 on paclitaxel efficacy was studied. For each part, cells were divided into four groups: negative control group, propofol group, paclitaxel group, and cotreatment group.

### 2.5 Bromodeoxyuridine (BrdU) incorporation assay

Cells (1.5 × 10^5^ cells/well) were dispensed in 6‐well plates and cultured with different treatments based on the above groups for 48 h. Then, BrdU (50 μg/mL) was added to the medium. After 6 h, the medium was removed, and the cells were washed with PBS and fixed with 4% paraformaldehyde for 30 min. DNA was denatured with 2 mol/L HCl for 5 min, the cell membrane was permeabilized by incubation with 0.2% Triton X‐100 for 20 min, and the cells were treated with 3% BSA for 1 h. The cells were rinsed three times with PBS between each step. The cells were incubated with an α‐BrdU antibody (mouse mAb, CST, 1:200) at 4°C overnight. Then, the cells were rewarmed at 37°C for 30 min and washed with PBS. The cells were incubated with the secondary antibody for 30 min and rinsed three times with PBS. The cells were stained with DAB and hematoxylin and then counted under a microscope (DMi8, Leica). The experiment was performed three times.

### 2.6 Flow cytometry assay

Cells (4 × 10^5^ cells/dish) were seeded in 60‐mm diameter dishes and incubated overnight for adherence. The cells were subjected to the above treatments for 48 h. Afterward, the cells were dissociated with 0.25% trypsin, washed with PBS and resuspended in 100 μL of 1 × binding buffer. The cells were stained with Annexin V‐FITC and propidium iodide (PI) solution for 15 min in the dark. Next, 400 μL of binding buffer was added to each group, and apoptosis was evaluated by a CytoFLEX flow cytometer (Beckman Coulter, USA). The assay was carried out three times.

### 2.7 Transwell assays

A total of 2 × 10^5^ cells/well were dispensed in 6‐well plates and incubated for attachment. the corresponding treatments were applied for 48 h, 2 × 10^4^ (for the migration assay) or 5 × 10^4^ (for the invasion assay) cells were collected in 100 μL of serum‐free medium and then added into the top chambers (Costar, USA), which contained a membrane with or without a coating of Matrigel basement membrane matrix (Biosciences, USA). A total of 700 μL of complete culture medium was added to the lower chambers. After 24 h, the cells were treated with 4% fixative, stained with 0.25% crystal violet (Beyotime, China) and observed under a microscope. The experiment was performed three times.

### 2.8 Western blot analysis

After the drug treatments were applied for 48 h, total cellular protein was extracted. The following protocol was described in our previous study.^14^ The primary antibodies were a rabbit anti‐stathmin 1 antibody, rabbit anti‐β‐tubulin antibody, and rabbit anti‐GAPDH antibody. The chemiluminescent bands were detected and visualized by a Bio‐Rad ChemiDocTM XRS system (Bio‐Rad, USA). The experiment was repeated three times.

### 2.9 Cell transfection

The stathmin 1 cDNA sequence was purchased from Changsha Youbio Biosciences Inc. and was inserted into the pCDH‐GFP + Puro vector to establish stathmin 1‐overexpressing CaSki cells. The lentivirus particles were collected after transfection of the corresponding vectors along with the auxiliary plasmids pMD2.G and psPAX2 into HEK293T cells. Then, CaSki cells were infected with the lentiviruses, and 2 μg/mL puromycin was utilized to select cells with stable stathmin 1 overexpression. Western blot was utilized to evaluate the efficiency of stathmin 1 overexpression.

### 2.10 Statistical analysis

SPSS 22.0 software was used to show the data as the mean and standard deviation (SD) values (IBM Corporation, USA). The Kolmogorov–Smirnov test was utilized to estimate the conformity of the data to a Gaussian distribution. Student's *t*‐test or Welch's *t*‐test was conducted to assess the significance of differences between two groups, and one‐way analysis of variance or the Mann–Whitney U test was conducted to determine the significance of multigroup differences. Two‐tailed *p* values of less than 0.05 were considered to indicate significant differences.

## 3 RESULTS

### 3.1 The effect of propofol and propofol combined with paclitaxel on the viability of cervical cancer cells

**FIGURE 1:**
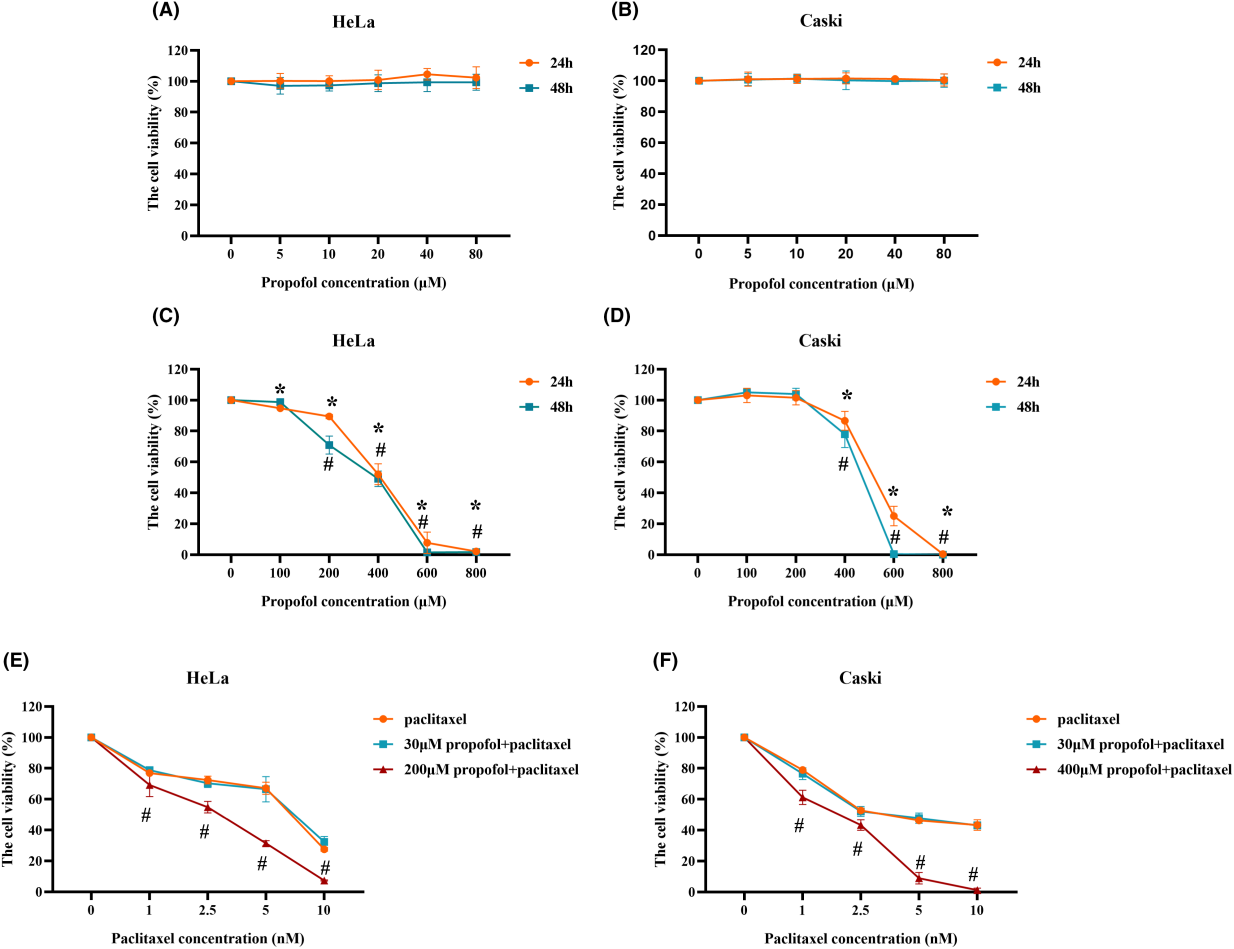
The effect of propofol and paclitaxel on the viability of HeLa and CaSki cells. (A, B) HeLa and CaSki cells were exposed to propofol at various concentrations commonly used in the clinic (0, 5, 10, 20, 40, and 80 μM) for 24 and 48 h; (C, D) HeLa and CaSki cells were exposed to propofol at high concentrations (0, 100, 200, 400, 600, and 800 μM) for 24 and 48 h; (E, F) HeLa and CaSki cells were exposed to 30 μM propofol or the IC30 of propofol (200 μM in HeLa and 400 μM in CaSki cells) in combination with various concentrations of paclitaxel (0, 1, 2.5, 5, and 10 nM) for 48 h. *p < 0.05 vs. the control group at 24 h, #*p* < 0.05 vs. the control group at 48 h.

Various clinically relevant concentrations of propofol displayed no effect on the viability of HeLa and CaSki cells after treatment for 24 or 48 h (Figure 1A,B). Propofol reduced HeLa cell viability in a dose‐dependent manner (Figure 1C). In CaSki cells, 100 and 200 μM propofol had no effect on viability, while 400, 600, and 800 μM propofol decreased cell viability (Figure 1D).

Propofol‐induced anesthesia usually occurs at a concentration of 4–6 mg/L, which is equivalent to 22–34 μM. Therefore, we chose 30 μM as the clinically relevant concentration in the follow‐up experiments. Cells were treated with 30 μM and the IC30 of propofol (200 μM for HeLa and 400 μM for CaSki cells) combined with various concentrations of paclitaxel for 48 h. The CCK‐8 assay results showed that 30 μM propofol had no influence on the cytotoxic effect of paclitaxel, while propofol at its IC30 enhanced the efficacy of paclitaxel in HeLa and CaSki cells (Figure 1E,F).

Subsequently, the CI values were calculated to evaluate the interaction between paclitaxel and propofol. As shown in Table 1, the IC30 of propofol and 5 or 10 nM paclitaxel had synergistic effect. Hence, 5 nM paclitaxel was chosen to combine with 30 μM and the IC30 of propofol in further experiments.

**TABLE 1:** The CI values of combination of propofol and paclitaxel.

Concentration of propofol	Cell	Concentration of paclitaxel	CI value
200	HeLa	1	1.09
		2.5	1.12
		5	0.85
		10	0.47
400	Caski	1	1.51
		2.5	1.37
		5	0.85
		10	0.69

### 3.2 The effect of propofol combined with paclitaxel on the proliferation of cervical cancer cells

**FIGURE 2:**
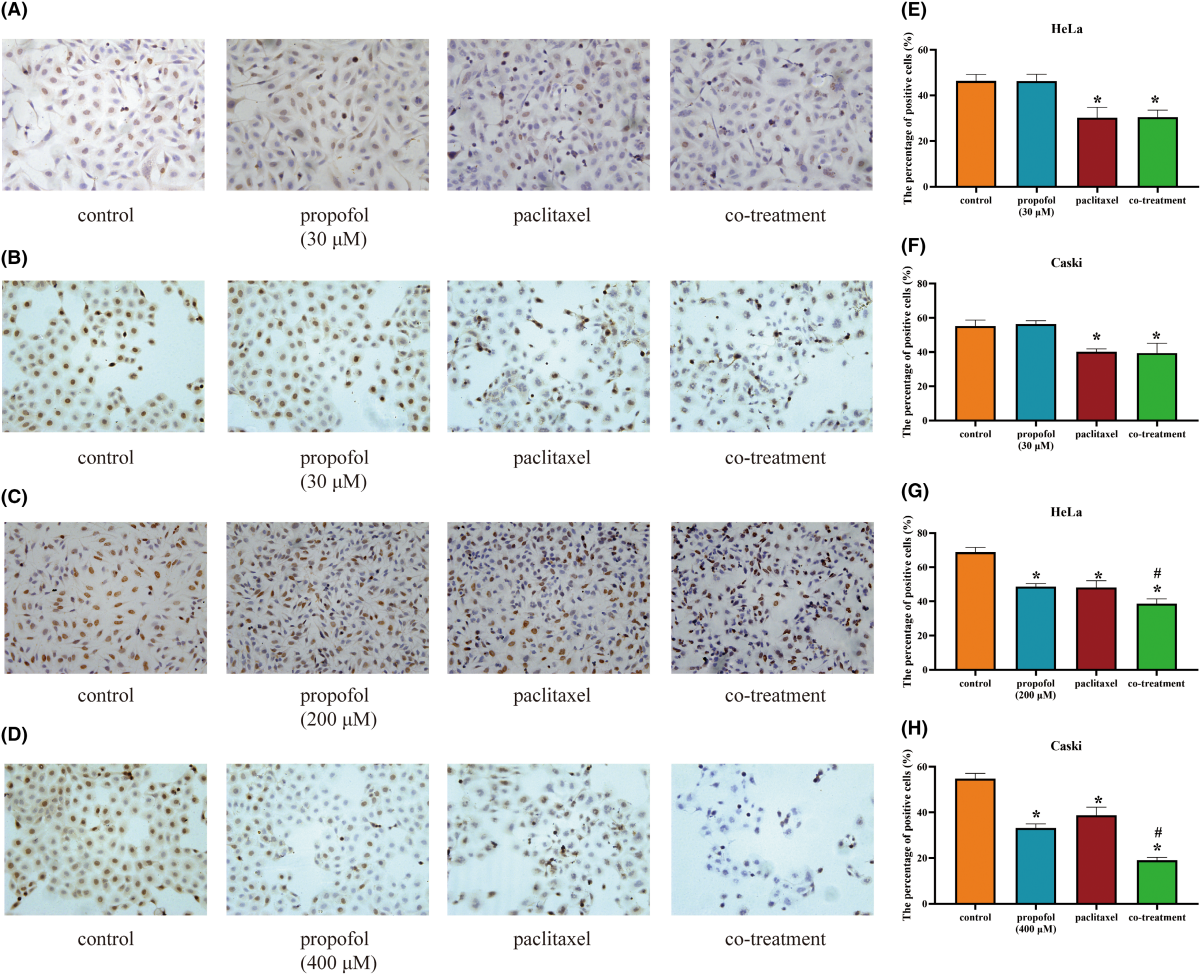
The effect of propofol and paclitaxel on the proliferation of HeLa and CaSki cells. (A, B) HeLa and CaSki cells were treated with the negative control (complete culture medium), propofol (30 μM), paclitaxel (5 nM) or the combination of propofol and paclitaxel. (C, D) HeLa and CaSki cells were treated with the control (complete culture medium), propofol (200 μM in HeLa and 400 μM in CaSki cells), paclitaxel (5 nM) or the combination of propofol and paclitaxel. The cell proliferation rates were assessed by a BrdU incorporation assay. (E–H) The bar graph shows the data from A–D. *p < 0.05 vs. the control group, #*p* < 0.05 vs. the paclitaxel group.

The results of the BrdU incorporation assay in HeLa and CaSki cells revealed that the proportions of positive cells in the paclitaxel groups were decreased compared with those in the corresponding control groups (*p* < 0.05). Propofol (30 μM) showed no effect on the efficacy of paclitaxel in HeLa and CaSki cells (Figure 2A,B). In addition, 200 μM propofol (HeLa cells) and 400 μM propofol (CaSki cells) suppressed cell growth and enhanced the cytotoxic effect of paclitaxel (Figure 2C,D *p*<0.05).

### 3.3 The effect of propofol combined with paclitaxel on apoptosis in cervical cancer cells

**FIGURE 3:**
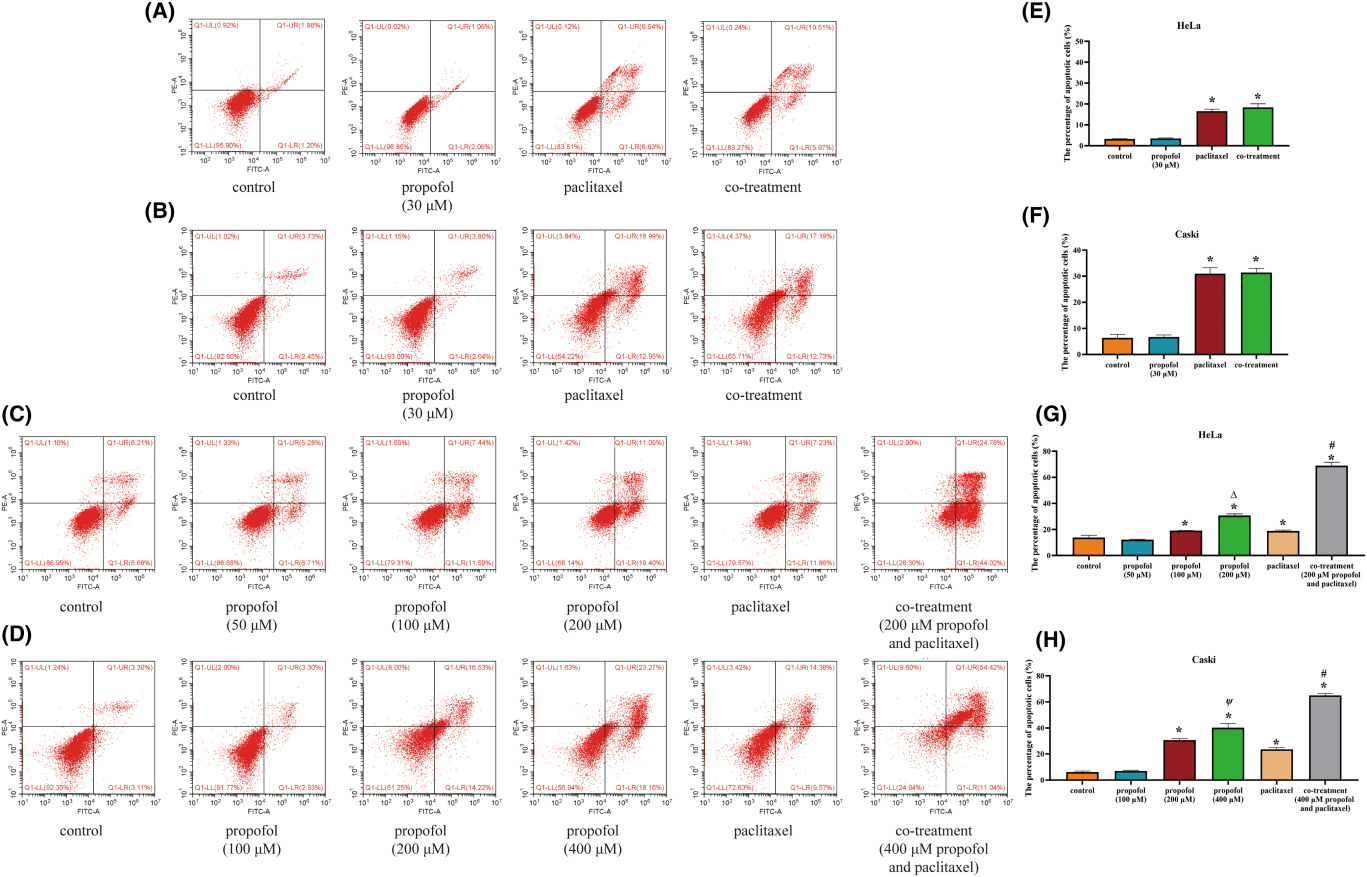
The effect of propofol and paclitaxel on apoptosis in HeLa and CaSki cells. (A, B) HeLa and CaSki cells were treated with the control (complete culture medium), propofol (30 μM), paclitaxel (5 nM) or the combination of propofol and paclitaxel. (C, D) HeLa and CaSki cells were treated with the control (complete culture medium), propofol (50, 100, and 200 μM in HeLa cells; 100, 200, and 400 μM in CaSki cells), paclitaxel (5 nM) or the combination of propofol and paclitaxel. (E–H) The bar graph shows the data from A–D. *p < 0.05 vs. the control group, #*p* < 0.05 vs. the paclitaxel group, ∆*p* < 0.05 vs. the 100 μM propofol group, ψ*p* < 0.05 vs. the 200 μM propofol group.

Annexin‐V/PI double staining was utilized to identify apoptotic cells. In sharp contrast to the control treatment, paclitaxel enhanced apoptosis in HeLa and CaSki cells (Figure 3, *p* < 0.05). However, 30 μM propofol did not influence the promoting effect of paclitaxel on apoptosis. As the concentration increased, propofol accelerated apoptosis in HeLa and CaSki cells and facilitated the apoptotic effect of paclitaxel on these cells (*p* < 0.05).

### 3.4 The effect of propofol combined with paclitaxel on the invasion and migration of cervical cancer cells

**FIGURE 4:**
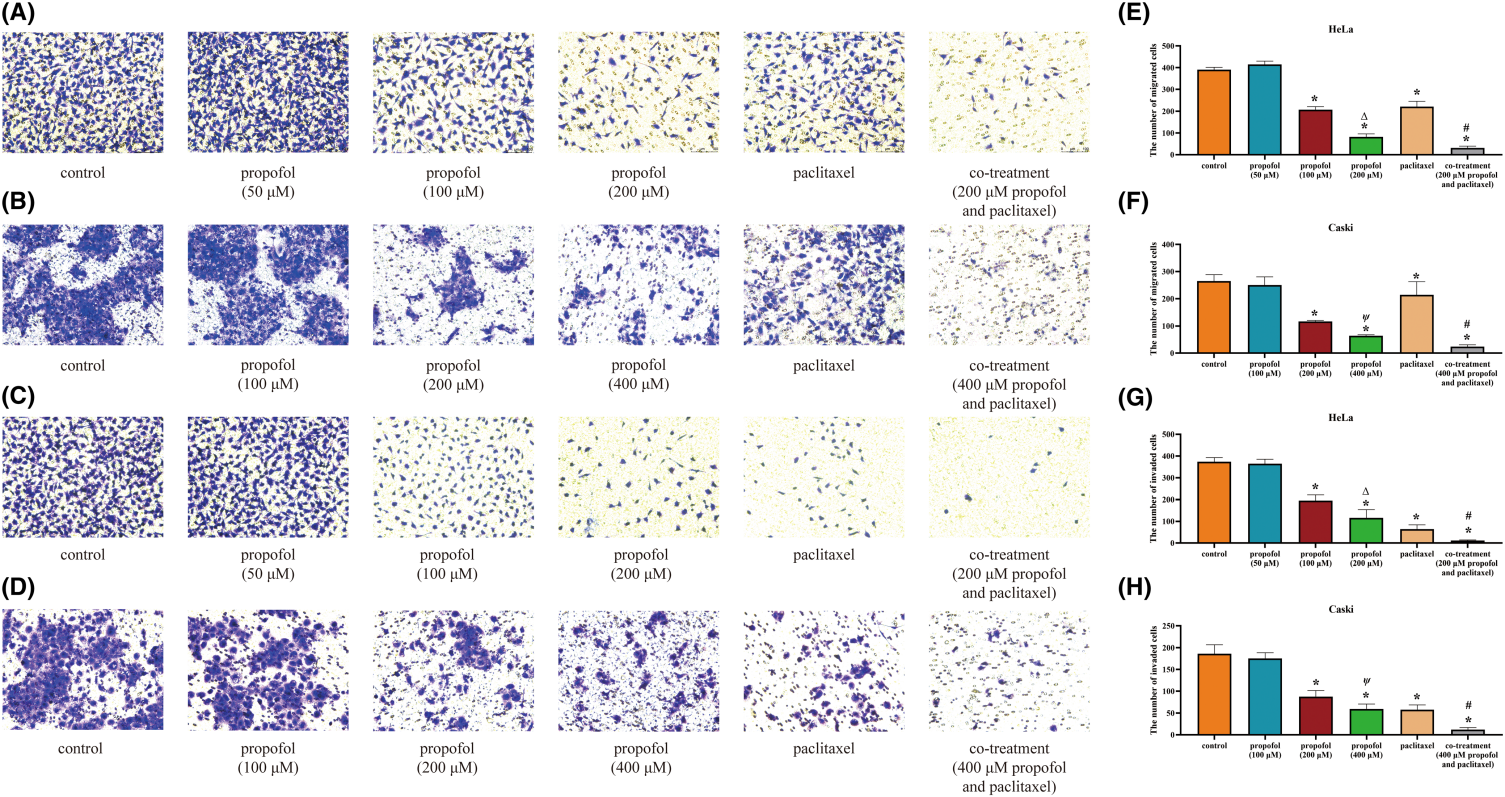
The effect of propofol and paclitaxel on the migration and invasion of HeLa and CaSki cells. (A, B) The effect on the migration of cervical cancer cells. HeLa and CaSki cells were treated with the control (complete culture medium), propofol (50, 100 and 200 μM in HeLa cells; 100, 200 and 400 μM in CaSki cells), paclitaxel (5 nM) or the combination of propofol and paclitaxel. (C, D) The effect on the invasion of cervical cancer cells. HeLa and CaSki cells were treated with the control (complete culture medium), propofol (50, 100 and 200 μM in HeLa cells; 100, 200 and 400 μM in CaSki cells), paclitaxel (5 nM) or the combination of propofol and paclitaxel. (E–H) The bar graph shows the data from A–D. *p < 0.05 vs. the control group, #*p* < 0.05 vs. the paclitaxel group, ∆*p* < 0.05 vs. the 100 μM propofol group, ψ*p* < 0.05 vs. the 200 μM propofol group.

Transwell assays were conducted to analyze the effect of the two drugs on cell migration and invasion. Propofol (100 and 200 μM for HeLa cells; 200 and 400 μM for CaSki cells) and paclitaxel (5 nM) decreased the numbers of migrated and invaded cells compared to those in the corresponding control groups (Figure 4). The migratory and invasive abilities in the 200 μM propofol group of HeLa cells and in the 400 μM propofol group of CaSki cells were lower than those in the 100 μM propofol group of HeLa cells and the 200 μM propofol group of CaSki cells, respectively (*p* < 0.05). Moreover, the combination of propofol and paclitaxel showed the greatest suppressive effect on the migration and invasion of cervical cancer cells (*p* < 0.05).

### 3.5 The effect of propofol combined with paclitaxel on the protein expression of β‐tubulin and stathmin 1 in cervical cancer cells

**FIGURE 5:**
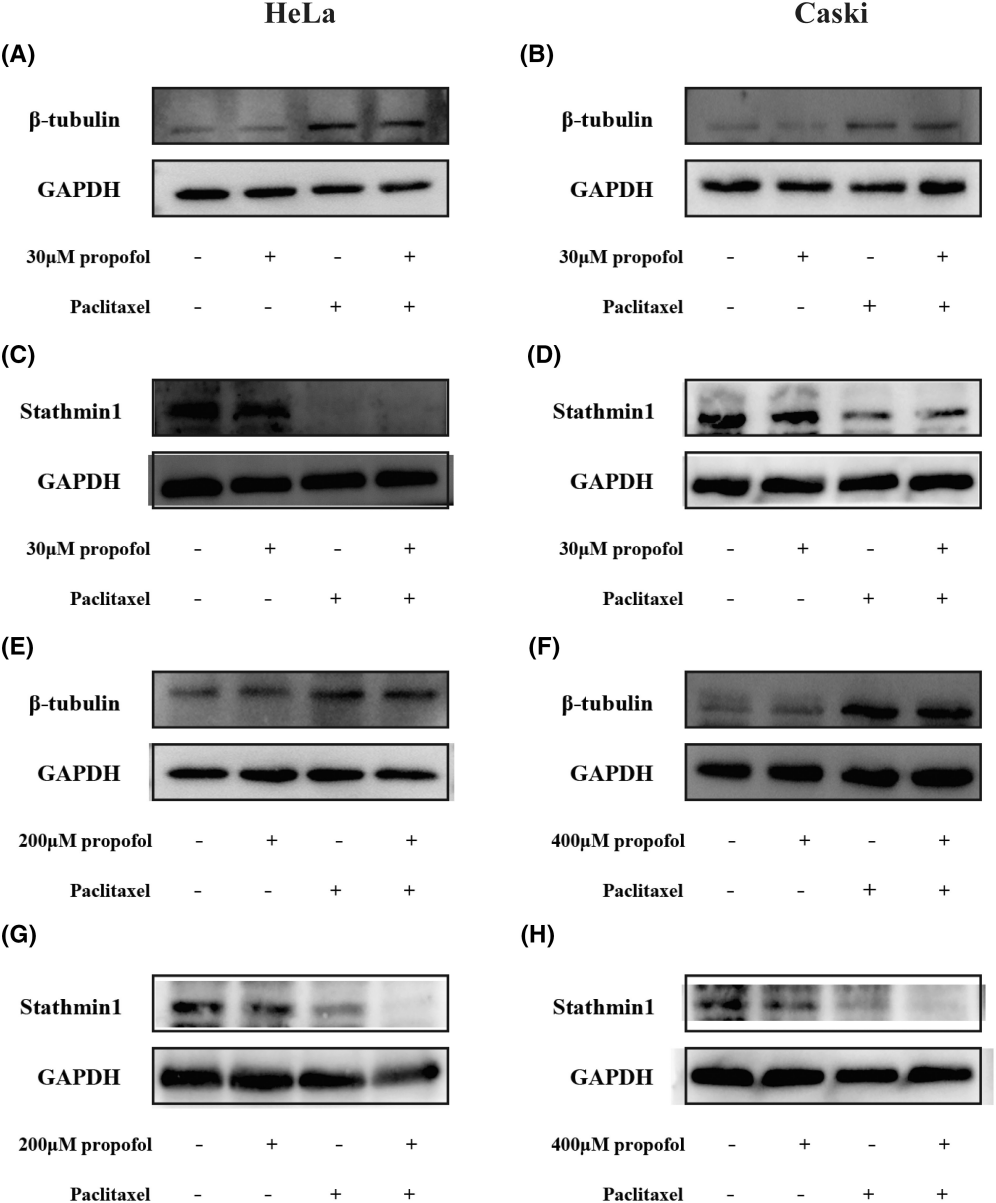
The effect of propofol and paclitaxel on the expression levels of β‐tubulin and stathmin 1 in cervical cancer cells. (A–D) HeLa and CaSki cells were treated with the control (complete culture medium), propofol (30 μM), paclitaxel (5 nM) or the combination of propofol and paclitaxel. (E–H) HeLa and CaSki cells were treated with the control (complete culture medium), propofol (200 μM in HeLa cells; 400 μM in CaSki cells), paclitaxel (5 nM) or the combination of propofol and paclitaxel.

Western blot analysis was conducted to measure the protein expression levels of β‐tubulin and stathmin 1 in HeLa and CaSki cells. Paclitaxel upregulated β‐tubulin expression but downregulated stathmin 1 expression in HeLa and CaSki cells (Figure 5). However, 30 μM propofol did not affect the expression of β‐tubulin protein or stathmin 1 protein in cervical cancer cells (Figure 5A–D). In addition, treatment with the IC30 of propofol (200 μM in HeLa and 400 μM in CaSki cells) did not influence the expression of β‐tubulin but downregulated the expression of stathmin 1 (Figure 5E–H). Propofol at its IC30 did not influence the effect of paclitaxel on the expression of β‐tubulin but amplified the inhibitory effect of paclitaxel on stathmin 1 expression in cancer cells.

### 3.6 The role of stathmin 1 in the effect of propofol on the efficacy of paclitaxel

**FIGURE 6:**
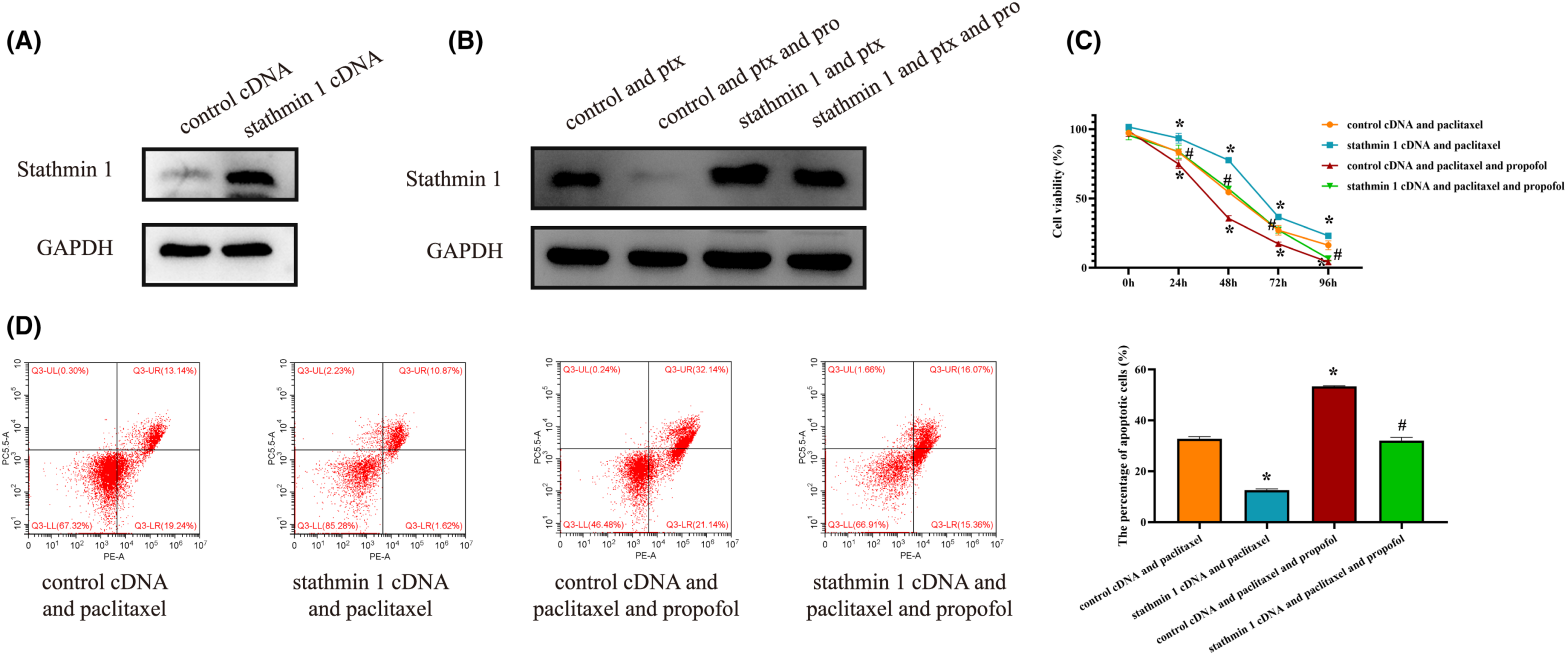
The role of stathmin 1 in the effect of propofol on the efficacy of paclitaxel. (A) The establishment of stathmin 1‐overexpressing CaSki cells. (B) The effect of propofol combined with paclitaxel on stathmin 1 expression. (C, D) The effect of paclitaxel combined with stathmin 1 overexpression and propofol on cell viability and apoptosis. *p < 0.05 vs. the control cDNA and paclitaxel group, #*p* < 0.05 vs. the stathmin 1 cDNA and paclitaxel group.

To investigate the role of stathmin 1 in the effect of propofol, we established stathmin 1‐overexpressing CaSki cells (Figure 6A) and found that propofol reduced the stathmin 1 protein level (Figure 6B). High expression of stathmin 1 weakened the inhibitory effect of paclitaxel on cell viability, while propofol treatment partially reversed this effect (Figure 6C *p*<0.05). In addition, stathmin 1 impaired the proapoptotic effect of paclitaxel, but the addition of propofol partially restored this effect of paclitaxel (Figure 6D *p*<0.05).

## 4 DISCUSSION

Anesthesia is an essential requirement in radical tumor resection surgery. To date, many studies have demonstrated that anesthetics could interfere with cancer development and chemoresistance.^15^ Our previous study showed that sevoflurane promoted the malignant behavior of cervical cancer cells but had no effect on cisplatin sensitivity.^16^ In addition, isoflurane upregulated the expression of miR‐216 to attenuate the malignant progression of colorectal cancer cells.^17^ Propofol is a widely used intravenous anesthetic, and increasing evidence has shown that at clinically relevant concentrations (10–100 μM), propofol significantly suppresses the malignant biological behaviors of cancer cells.^18^, ^19^, ^20^ Du et al.^21^ demonstrated that 30–100 μM propofol reduced the viability of CaSki and SiHa cells in a dose‐ and time‐dependent manner. However, our study demonstrated that 0–100 μM propofol had no effect on cell viability after treatment for 24 and 48 h and that 30 μM propofol did not affect the malignancy of HeLa and CaSki cells after treatment for 48 h.

Mitosis is a necessary pathway for cancer cell proliferation. The normal dynamic balance of microtubule polymerization and depolymerization is needed during mitosis to help chromosomes move to the poles via the tension generated by microtubule depolymerization and the thrust generated by microtubule polymerization.^22^ Paclitaxel, considered as a microtubule stabilizer, promotes the formation of tubulin dimers and stabilizes existing microtubules, and hinders their degradation to advance microtubule assembly.^23^ Owing to the excessive stability of microtubules, the cell cycle is blocked in the late G2 stage, and cell replication is suppressed.^24^ β‐Tubulin is bound strongly by paclitaxel to stabilize microtubule filaments.^25^ A study demonstrated that β‐tubulin mutations could increase the chemoresistance of cancer cells to paclitaxel.^26^ In the present study, paclitaxel treatment increased the level of β‐tubulin in cervical cancer cells. β‐Tubulin expression was not changed after propofol treatment, and the β‐tubulin protein expression level in the cotreatment group was not different from that in the paclitaxel group, indicating that propofol had no effect on β‐tubulin expression.

A phosphorylated protein commonly found in the cytoplasm in vertebrates, the stathmin 1 protein impedes the assembly and induces the disassembly of microtubules to take part in the process of mitosis.^27^ Various studies have shown that the stathmin 1 protein plays a pivotal role in the evolution of cancer.^28^, ^29^, ^30^ For example, the expression of stathmin 1 was negatively related to the degree of tumor differentiation, and a high level of stathmin 1 tended to indicate distant metastasis in pancreatic cancer.^31^ Due to the mechanism of paclitaxel in cancer cells, stathmin 1 is a potential drug target of paclitaxel. A study showed that downregulation of stathmin 1 reduced the malignancy of lung cancer cells and decreased the chemoresistance of cancer cells to paclitaxel.^32^ In patients with endometrial cancer, stathmin 1 was identified as a biomarker for unfavorable survival, but paclitaxel treatment might improve the outcomes of patients with high levels of stathmin 1 expression.^33^ The combination of paclitaxel treatment and silencing of stathmin 1 enhanced the tumoricidal effect compared with that of paclitaxel alone, and paclitaxel suppressed the expression of stathmin 1 in nasopharyngeal carcinoma.^34^ In the present research, paclitaxel decreased the expression of stathmin 1, and propofol at high concentrations enhanced the effect of paclitaxel on stathmin 1 expression in cervical cancer cells, indicating that propofol and paclitaxel synergistically suppress the malignancy of cancer cells by downregulating the expression of stathmin 1.

There are some other mechanisms underlying the effect of propofol on the efficacy of paclitaxel. It was reported that propofol might reduce SLUG expression to weaken the proapoptotic effect of paclitaxel in ovarian cancer.^35^ Yang et al.^6^ found that propofol elevated paclitaxel sensitivity by modulating HOTAIR expression. In addition, a study demonstrated that propofol enhanced the tumoricidal ability of paclitaxel partially by inducing ferroptosis.^36^, ^37^ In the present study, propofol might inhibit stathmin 1 expression to enhance the efficacy of paclitaxel.

In conclusion, propofol at high concentrations enhanced the cytotoxicity of paclitaxel, resulting in reduced malignancy of cervical cancer cells, by downregulating stathmin 1 expression, while propofol at clinically relevant concentrations had no effect on the efficacy of paclitaxel in cervical cancer cells; therefore, propofol at clinically relevant concentrations might not affect the response to paclitaxel in patients with recurrent or advanced cervical cancer.

## AUTHOR CONTRIBUTIONS

**Yanshan Jin:** Conceptualization (lead); formal analysis (lead); methodology (equal); writing – original draft (equal); writing – review and editing (lead). **Shangdan Xie:** Conceptualization (equal); data curation (equal); methodology (equal); writing – original draft (equal). **Bo Sheng:** Investigation (equal); writing – review and editing (equal). **Mei Chen:** Funding acquisition (equal); Formal analysis (equal); writing – review and editing (equal). **Xueqiong Zhu:** Funding acquisition (lead); resources (l); supervision (lead).

## CONFLICT OF INTEREST STATEMENT

The authors have declared no conflicts of interest.

## Data Availability

The data in the present study are available from the corresponding author on reasonable request.

## References

[1] Fan J , Zhou Q , Li Y , et al. Profiling of long non‐coding RNAs and mRNAs by RNA‐sequencing in the hippocampi of adult mice following propofol sedation. Front Mol Neurosci. 2018;11:91. 29628875 10.3389/fnmol.2018.00091 PMC5876304

[2] Miyahara T , Adachi N , Seki T , et al. Propofol induced diverse and subtype‐specific translocation of PKC families. J Pharmacol Sci. 2018;137(1):20‐29. 29699771 10.1016/j.jphs.2018.03.008

[3] Zhang H , Tan M , Zhang J , Han X , Ma Y . Propofol inhibits thyroid cancer cell proliferation, migration, and invasion by suppressing SHH and PI3K/AKT signaling pathways via the miR‐141‐3p/BRD4 Axis. J Health c Eng. 2021;2021:2704753. 10.1155/2021/2704753 PMC8702329 34956562

[4] Ye LL , Cheng ZG , Cheng XE , Huang YL . Propofol regulates miR‐1‐3p/IGF1 axis to inhibit the proliferation and accelerates apoptosis of colorectal cancer cells. Toxicol Res (Camb). 2021;10(4):696‐705. 34745557 10.1093/toxres/tfab047 PMC8561266

[5] Gao L , Zhang X . Propofol enhances the lethality of cisplatin on liver cancer cells by up‐regulating miR‐195‐5p. Tissue Cell. 2021;74:101680. 34808429 10.1016/j.tice.2021.101680

[6] Yang X , Qin J , Gong C , Yang J . Propofol enhanced the cell sensitivity to paclitaxel (PTX) in prostatic cancer (PC) through modulation of HOTAIR. Genes Genomics. 2021;43(7):807‐814. 33893626 10.1007/s13258-021-01093-0

[7] Siegel RL , Miller KD , Fuchs HE , Jemal A . Cancer statistics, 2021. CA Cancer J Clin. 2021;71(1):7‐33. 33433946 10.3322/caac.21654

[8] Greggi S , Casella G , Scala F , Falcone F , Visconti S , Scaffa C . Surgical management of early cervical cancer: when is laparoscopic appropriate? Curr Oncol Rep. 2020;22(1):7. 31989322 10.1007/s11912-020-0876-1

[9] Duenas‐Gonzalez A , Gonzalez‐Fierro A . Pharmacodynamics of current and emerging treatments for cervical cancer. Expert Opin Drug Metab Toxicol. 2019;15(8):671‐682. 31340683 10.1080/17425255.2019.1648431

[10] Hill EK . Updates in cervical cancer treatment. Clin Obstet Gynecol. 2020;63(1):3‐11. 31815773 10.1097/GRF.0000000000000507

[11] Della Corte L , Barra F , Foreste V , et al. Advances in paclitaxel combinations for treating cervical cancer. Expert Opin Pharmacother. 2020;21(6):663‐677. 32037907 10.1080/14656566.2020.1724284

[12] Dan VM , Raveendran RS , Baby S . Resistance to intervention: paclitaxel in breast cancer. Mini Rev Med Chem. 2021;21(10):1237‐1268. 33319669 10.2174/1389557520999201214234421

[13] Chou TC . Theoretical basis, experimental design, and computerized simulation of synergism and antagonism in drug combination studies. Pharmacol Rev. 2006;58(3):621‐681. 16968952 10.1124/pr.58.3.10

[14] Xu Y , Pan S , Chen H , Qian H , Wang Z , Zhu X . MEX3A suppresses proliferation and EMT via inhibiting Akt signaling pathway in cervical cancer. Am J Cancer Res. 2021;11(4):1446‐1462. 33948367 PMC8085868

[15] Zhang W , Xue F , Xie S , Chen C , Li J , Zhu X . Isoflurane promotes proliferation of squamous cervical cancer cells through mTOR‐histone deacetylase 6 pathway. Mol Cell Biochem. 2021;476(1):45‐55. 32833118 10.1007/s11010-020-03884-7 PMC7867516

[16] Xue F , Xu Y , Song Y , Zhang W , Li R , Zhu X . The effects of sevoflurane on the progression and cisplatinum sensitivity of cervical cancer cells. Drug des Devel Ther. 2019;13:3919‐3928. 10.2147/DDDT.S219788 PMC6873969 31819366

[17] Cai Z , Suo L , Huang Z . Isoflurane suppresses proliferation, migration, and invasion and facilitates apoptosis in colorectal cancer cells through targeting miR‐216. Front Med (Lausanne). 2021;8:658926. 34458282 10.3389/fmed.2021.658926 PMC8385302

[18] Gao J , Ding C , Zhou J , et al. Propofol suppresses lung cancer tumorigenesis by modulating the circ‐ERBB2/miR‐7‐5p/FOXM1 axis. Thorac Cancer. 2021;12(6):824‐834. 33506582 10.1111/1759-7714.13856 PMC7952809

[19] Zhang J , Zhang D , Wu GQ , Feng ZY , Zhu SM . Propofol inhibits the adhesion of hepatocellular carcinoma cells by upregulating microRNA‐199a and downregulating MMP‐9 expression. Hepatobiliary Pancreat Dis Int. 2013;12(3):305‐309. 23742776 10.1016/s1499-3872(13)60048-x

[20] Sun H , Gao D . Propofol suppresses growth, migration, and invasion of A549 cells by downregulation of miR‐372. BMC Cancer. 2018;18(1):1252. 30547768 10.1186/s12885-018-5175-y PMC6295097

[21] Du XT , Wang XY , Zheng YH , Liu DP . Propofol suppresses the growth and invasion of cervical carcinoma cells by inhibiting MIR155HG. Aging (Albany NY). 2021;13(21):24464‐24475. 34775376 10.18632/aging.203697 PMC8610141

[22] Barisic M , Maiato H . The tubulin code: a navigation system for chromosomes during mitosis. Trends Cell Biol. 2016;26(10):766‐775. 27344407 10.1016/j.tcb.2016.06.001 PMC6398581

[23] Farrar MC , Jacobs TF . Paclitaxel. *StatPearls* ; 2021.

[24] Manfredi JJ , Horwitz SB . Taxol: an antimitotic agent with a new mechanism of action. Pharmacol Ther. 1984;25(1):83‐125. 6149569 10.1016/0163-7258(84)90025-1

[25] Amaya C , Luo S , Baigorri J , Baucells R , Smith ER , Xu XX . Exposure to low intensity ultrasound removes paclitaxel cytotoxicity in breast and ovarian cancer cells. BMC Cancer. 2021;21(1):981. 34470602 10.1186/s12885-021-08722-7 PMC8408969

[26] Wang W , Zhang H , Wang X , et al. Novel mutations involving betaI‐, betaIIA‐, or betaIVB‐tubulin isotypes with functional resemblance to betaIII‐tubulin in breast cancer. Protoplasma. 2017;254(3):1163‐1173. 27943021 10.1007/s00709-016-1060-1

[27] Belletti B , Baldassarre G . Stathmin: a protein with many tasks. New biomarker and potential target in cancer. Expert Opin Ther Targets. 2011;15(11):1249‐1266. 21978024 10.1517/14728222.2011.620951

[28] Liu J , Li J , Wang K , et al. Aberrantly high activation of a FoxM1‐STMN1 axis contributes to progression and tumorigenesis in FoxM1‐driven cancers. Signal Transduct Target Ther. 2021;6(1):42. 33526768 10.1038/s41392-020-00396-0 PMC7851151

[29] Dos Santos PB , Lima K , Kremer JL , et al. Stathmin 1 is highly expressed and associated with survival outcome in malignant adrenocortical tumours. Invest New Drugs. 2020;38(3):899‐908. 31441020 10.1007/s10637-019-00846-9

[30] Liu P , Yu J , Tian X , et al. The effect of downregulation of Stathmin gene on biological behaviors of U373 and U87‐MG glioblastoma cells. Biol Res. 2018;51(1):16. 29880026 10.1186/s40659-018-0160-0 PMC5992777

[31] Suzuki K , Watanabe A , Araki K , et al. High STMN1 expression is associated with tumor differentiation and metastasis in clinical patients with pancreatic cancer. Anticancer Res. 2018;38(2):939‐944. 29374725 10.21873/anticanres.12307

[32] Bao P , Yokobori T , Altan B , et al. High STMN1 expression is associated with cancer progression and chemo‐resistance in lung squamous cell carcinoma. Ann Surg Oncol. 2017;24(13):4017‐4024. 28933054 10.1245/s10434-017-6083-0

[33] Reyes HD , Miecznikowski J , Gonzalez‐Bosquet J , et al. High stathmin expression is a marker for poor clinical outcome in endometrial cancer: an NRG oncology group/gynecologic oncology group study. Gynecol Oncol. 2017;146(2):247‐253. 28532857 10.1016/j.ygyno.2017.05.017 PMC5526627

[34] Wu Y , Tang M , Wu Y , et al. A combination of paclitaxel and siRNA‐mediated silencing of Stathmin inhibits growth and promotes apoptosis of nasopharyngeal carcinoma cells. Cell Oncol (Dordr). 2014;37(1):53‐67. 24306928 10.1007/s13402-013-0163-3 PMC13004449

[35] Wang P , Chen J , Mu LH , Du QH , Niu XH , Zhang MY . Propofol inhibits invasion and enhances paclitaxel‐ induced apoptosis in ovarian cancer cells through the suppression of the transcription factor slug. Eur Rev Med Pharmacol Sci. 2013;17(13):1722‐1729. 23852894

[36] Sun C , Liu P , Pei L , Zhao M , Huang Y . Propofol inhibits proliferation and augments the anti‐tumor effect of doxorubicin and paclitaxel partly through promoting Ferroptosis in triple‐negative breast cancer cells. Front Oncol. 2022;12:837974. 35419287 10.3389/fonc.2022.837974 PMC8996258

[37] Zhao MY , Liu P , Sun C , Pei LJ , Huang YG . Propofol augments paclitaxel‐induced cervical cancer cell ferroptosis in vitro. Front Pharmacol. 2022;13:816432. 35517791 10.3389/fphar.2022.816432 PMC9065257

